# The Rising Tide of Antibiotic Resistance: A Study on Extended‐Spectrum Beta‐Lactamase and Carbapenem‐Resistant *Escherichia coli* and *Klebsiella pneumoniae*

**DOI:** 10.1002/jcla.25081

**Published:** 2024-06-17

**Authors:** Hasan Ejaz, Muhammad Usman Qamar, Aisha Farhana, Sonia Younas, Alia Batool, Durreshahwar Lone, Muhammad Atif, Mashael W. Alruways, Muharib Alruwaili, Ismail Hamad, Samy Selim, Bi Bi Zainab Mazhari, Ali Farooq, Kashaf Junaid

**Affiliations:** ^1^ Department of Clinical Laboratory Sciences, College of Applied Medical Sciences Jouf University Sakaka Saudi Arabia; ^2^ Institute of Microbiology, Faculty of Life Sciences Government College University Faisalabad Faisalabad Pakistan; ^3^ Division of Infectious Diseases, Department of Medicine Geneva University Hospitals and Medical School Geneva Switzerland; ^4^ HKU‐Pasteur Research Pole, School of Public Health, LKS Faculty of Medicine The University of Hong Kong Hong Kong China; ^5^ Department of Pathology Fatima Memorial Hospital College of Medicine and Dentistry Lahore Pakistan; ^6^ Department of Clinical Laboratory Sciences, College of Applied Medical Sciences Shaqra University Shaqra Saudi Arabia; ^7^ Department of Clinical Laboratory Sciences, College of Applied Medical Sciences Jouf University Qurayyat Saudi Arabia; ^8^ Department of Internal Medicine T.H.Q. Hospital Pirmahal Punjab Pakistan; ^9^ School of Biological and Behavioural Sciences, Queen Mary University of London London UK

**Keywords:** antimicrobial resistance genes, beta‐lactam antibiotics, carbapenem‐resistant Enterobacterales, drug resistance prevalence, ESBL

## Abstract

**Background:**

The global spread of extended‐spectrum beta‐lactamase (ESBL)‐producing and carbapenem‐resistant Enterobacterales (CRE) poses a significant concern. Acquisition of antimicrobial resistance genes leads to resistance against several antibiotics, limiting treatment options. We aimed to study ESBL‐producing and CRE transmission in clinical settings.

**Methods:**

From clinical samples, 227 ESBL‐producing and CRE isolates were obtained. The isolates were cultured on bacterial media and confirmed by VITEK 2. Antibiograms were tested against several antibiotics using VITEK 2. The acquired resistance genes were identified by PCR.

**Results:**

Of the 227 clinical isolates, 145 (63.8%) were *Klebsiella pneumoniae* and 82 (36.1%) were *Escherichia coli*; 76 (33.4%) isolates were detected in urine, 57 (25.1%) in pus swabs, and 53 (23.3%) in blood samples. A total of 58 (70.7%) ESBL‐producing *E. coli* were resistant to beta‐lactams, except for carbapenems, and 17.2% were amikacin‐resistant; 29.2% of *E. coli* isolates were resistant to carbapenems. A total of 106 (73.1%) ESBL‐producing *K. pneumoniae* were resistant to all beta‐lactams, except for carbapenems, and 66.9% to ciprofloxacin; 38 (26.2%) *K. pneumoniae* were resistant to carbapenems. Colistin emerged as the most effective antibiotic against both bacterial types. Twelve (20.6%) *E. coli* isolates were positive for *bla*_CTX‐M_, 11 (18.9%) for *bla*_TEM_, and 8 (33.3%) for *bla*_NDM_. Forty‐six (52.3%) *K. pneumoniae* isolates had *bla*_CTX‐M_, 27 (18.6%) *bla*_TEM_, and 26 (68.4%) *bla*_NDM_.

**Conclusion:**

This study found a high prevalence of drug‐resistant ESBL‐producing and CRE, highlighting the need for targeted antibiotic use to combat resistance.

## Graphical abstract

This study investigates the transmission of extended‐spectrum beta‐lactamase‐producing and carbapenem‐resistant *Klebsiella pneumoniae* and *Escherichia coli*. Widespread resistance among these pathogens underscores the need for a comprehensive analysis of antibiotic resistance patterns to mitigate resistance effectively.

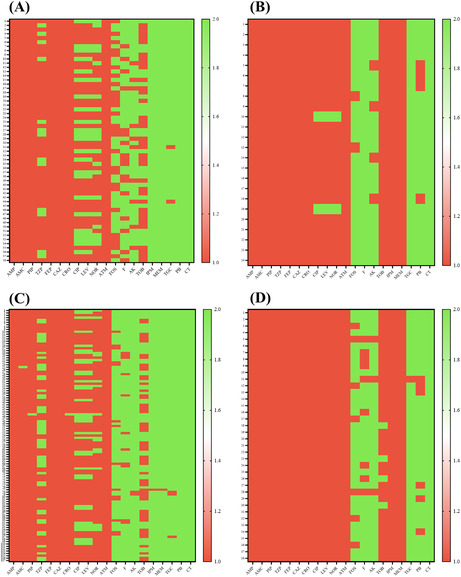

## 1 Introduction

The widespread emergence of antimicrobial resistance (AMR) can be attributed to the excessive and inappropriate use of antibiotics, as well as inadequate infection control strategies like immunization [1]. AMR, also referred to as the “silent pandemic,” is progressively becoming more prevalent and is of great concern globally. It is a significant problem in Pakistan, a country categorized as a lower‐middle income country (LMIC) [2]. The mortality rate worldwide in 2019 due to AMR was a matter of significant concern, as it reached 4.95 million deaths, with 1.27 million directly attributed to infections [3]. This would result in a significant increase, with an estimated 10 million deaths by 2050. The World Health Organization (WHO) expressed its worry in 2017 regarding the heightened levels of mortality [4]. The WHO's statement raises concerns about the current use of antibiotics, highlighting that a considerable number of them are derived from the existing WHO classes of antibiotics and have a limited duration of effectiveness. Pakistan has had difficulties effectively addressing growing prevalence of AMR [5]. Between the years 2000 and 2015, Pakistan experienced a significant rise of 65% in the utilization of antibiotics. The use of antibiotics under the antibiotic “Watch” group had a significant increase of 61.5% between 2014 and 2018, hence giving rise to concerns [6].

The emergence of extended‐spectrum beta‐lactamase (ESBL)‐producing and carbapenem‐resistant Enterobacterales (CRE), particularly *Escherichia coli* and *Klebsiella pneumoniae*, has garnered considerable interest due to their association with a wide range of adverse outcomes in hospitalized patients, posing a significant risk to their well‐being [7, 8]. These microorganisms generate a diverse array of diseases, notably nosocomial infections [9]. According to the WHO, there has been a gradual increase in the worldwide prevalence of this bacterial taxon, leading to its recognition as pathogens with high priority in terms of antibiotic resistance [10]. These pathogens produce resistance to medically important “Access” and “Watch” groups of antibiotics and leave limited treatment options [11]. The global per capita consumption of “Watch” antibiotics climbed by 90.9% between 2000 and 2015, whereas the consumption of “Access” antibiotics increased by 26.2% during the same period. A greater rise in antibiotic use has been more pronounced in LMICs than in high‐income countries [12].

Antibiotic resistance is primarily due to the acquisition of ESBL genes, mainly *bla*_CTX‐M_, *bla*_TEM_, and *bla*_SHV_, which hydrolyze the cephalosporin class of antibiotics [13] and carbapenem‐resistant (CR) genes, notably *bla*_NDM_, *bla*_VIM_, and *bla*_IMP_, which hydrolyze beta‐lactam antibiotics [14]. Therefore, this study was structured to explore the spread and coexistence of pathogens harboring antimicrobial‐resistant genes (ARGs) within clinical environments.

## 2 Materials and Methods

### 2.1 Collection of Bacteria

This study adhered to the ethical standards set by the Declaration of Helsinki and did not involve any human or animal subjects [15]. The 227 leftover clinical isolates of *E. coli* and *K. pneumoniae*, collected from various sources including blood, swabs, urine, and sputum, were obtained from clinical settings in Pakistan. This study specifically focused on analyzing *E. coli* and *K. pneumoniae*, excluding other types of bacteria. The analysis incorporated the clinical data of patients, encompassing variables such as gender, age, and the origin of specimens. The blood culture samples were inoculated into blood culture bottles and thereafter subjected to a maximum processing duration of 5 days in an automated BACTEC blood culture system (BD, USA). The remaining clinical specimens, including body fluids, pus, sputum, and midstream urine, were obtained following established protocols. The specimens underwent processing within the confines of the pathology laboratory.

### 2.2 Processing of the Isolates

Depending on the type of clinical sample, different types of agar were used for culture, such as nutrient agar, blood agar, McConkey's medium, and CLED medium. The plates were incubated at 37°C to monitor the growth of microbes. Gram staining, colony shape, and growth characteristics were initially used to identify the isolates. An automated VITEK 2 system (bioMérieux, France) equipped with GN cards was used to verify the identity of the isolates [16]. In clinical microbiology, the VITEK 2 system is crucial because it speeds up the process of identifying infectious pathogens and testing them for susceptibility. The biochemical and metabolic signatures, for example, substrate consumption, acid generation, enzyme activity, and other physiochemical features, of the microorganisms were examined.

### 2.3 Antibiogram of the Clinical Isolates

Antimicrobial susceptibility testing (AST) of each isolate was performed using the VITEK 2 compact equipment (BioMérieux, France). In addition to microbiological identification, the VITEK 2 system is frequently employed for AST analysis. The assessment of bacterial and fungal susceptibility to various antimicrobial treatments requires AST, which is a critical component of clinical microbiology. Each antibiotic used in this experiment was classified as “Access,” “Watch,” or “Reserve” according to the AWaRe classification of the WHO. “Access” antibiotics applied to the isolates were ampicillin, piperacillin/tazobactam, amoxicillin/clavulanic acid, amikacin, and nitrofurantoin. Antibiotics included in the “Watch” group were fosfomycin, ceftriaxone, ciprofloxacin, levofloxacin, norfloxacin, imipenem, and meropenem. Tigecycline and polymyxin B and E were the only “Reserve” antibiotics employed against the isolates in this investigation. Antimicrobial susceptibility was interpreted in accordance with established standard guidelines [17].

### 2.4 Phenotypic Identification of ESBLs

The laboratory assays used for the phenotypic identification of ESBL in bacteria evaluated the ability of bacteria to hydrolyze extended‐spectrum beta‐lactam antibiotics. The double‐disc synergy test (DDST) was employed to identify ESBL in Gram‐negative rods (GNRs). The DDST involved the placement of two antibiotic disks onto a Mueller Hinton agar (MHA) plate that had been infected with the test bacterium at a concentration of 0.5 McFarland. The second disk in the experiment consisted of a combination of third‐generation cephalosporins (such as ceftriaxone or ceftazidime) and clavulanic acid, which acted as a beta‐lactamase inhibitor. The MHA plates were incubated at 37°C for the duration of one night. The identification of ESBL was established when a discernible increase in the zone of inhibition surrounding the combination disk (including clavulanate) was detected in contrast to the zone surrounding the disk containing only cephalosporin. Producers that satisfied the designated criteria were categorized as ESBL [18].

### 2.5 Phenotypic Detection of Carbapenem‐Resistant Enterobacterales

Enterobacterales phenotypes resistant to carbapenem antibiotics, often used as a last resort, can be detected using CRE testing in the laboratory. Imipenem and meropenem resistance (minimum inhibitory concentration [MIC] >1 μg/mL) and CRE phenotyping were performed on all Enterobacterales clinical isolates [17].

### 2.6 Molecular Detection of Genes

Genomic DNA was extracted from clinical isolates utilizing a bacterial genomic DNA kit (Thermo Scientific, USA). NanoDrop spectrophotometer absorbance readings at 260 and 280 nm were used to determine the DNA sample's purity. We employed a previously established primer sequence for gene detection (Table 1). Following these detailed protocols, the existence of *bla*_NDM_ was confirmed. The sample was heated to 95°C for 1 min to begin the denaturation process and then heated again for 45 s. After that, the sample was annealed for 45 s at different annealing temperatures for each primer (50°C, 52°C, 56°C, 58°C, and 60°C) and then for 1 min at 72°C. The sample was then kept in an oven at 72°C for an additional 5 min after running the polymerase chain reaction (PCR) results on 1% agarose gels with a 1 kb DNA ladder (Thermo Scientific, USA). We used GraphPad Prism 9 for the data analysis.

**TABLE 1:** Sequence of primers used in the study.

Gene	Primers (5′–3′)	Annealing temperature	Reference
*bla*_CTX‐M_‐F	ATGTGCAGYACCAGTAARGTKATGGC	62°C	[19]
*bla*_CTX‐M_‐R	TGGGTRAARTARGTSACCAGAAYCAGCGG		
*bla*_TEM_‐F	CGCCGCATACACTATTCTCAGAATGA	62°C	
*bla*_TEM_‐R	ACGCTCACCGGCTCCAGATTTAT		
*bla*_NDM_‐F	ATGGAATTGCCCAATATTATGCAC	58°C	
*bla*_NDM_‐R	TCAGCGCAGCTTGTCGGC		
*bla*_SHV_‐F	ATTTGTCGCTTCTTTACTCGCC	56°C	[7]
*bla*_SHV_‐R	TTCACCACCATCATTACCGACC		
*bla*_OXA‐48_‐F	TTGGTGGCATCGATTATCGG	52°C	[20]
*bla*_OXA‐48_‐R	GAGCACTTCTTTTGTGATGGC		
*bla*_IMP_‐F	GGAATAGAGTGGCTTAAYTC	50°C	
*bla*_IMP_‐R	TCGGTTTAAYAAAACAACCACC		
*bla*_VIM_‐F	GATGGTGTTTGGTCGCATA	50°C	
*bla*_VIM_‐R	CGAATGCGCAGCACCAG		
*mcr‐1*‐F	AGTCCGTTTGTTCTTGTGGC	58°C	[21]
*mcr‐1*‐R	AGATCCTTGGTCTCGGCTTG		

## 3 Results

### 3.1 Clinical Profile of Patients

Of the 227 clinical isolates from different samples, 145 (63.8%) were *K. pneumoniae* isolates and 82 (36.1%) were *E. coli* isolates. Most of the isolates (42; 18.0%) were from children younger than 10 years of age, followed by people aged 31–40 years (35; 15.4%) and 21–30 years (31; 13.6%). The male‐to‐female ratio was 1:0.9, with most isolates coming from males (116; 51.1%) compared with females. Table 2 shows that 76 (33.4%) of the isolates were found in urine samples from clinical settings, followed by 57 (25.1%) from pus swabs, 53 (23.3%) from blood culture, and 21 (9.2%) from catheter tips.

**TABLE 2:** Demographic and clinical profile of patients with distribution of sample types and isolates.

Clinical information	Frequency	Percentage
**Age range (1–89 years)**		
0–10	42	18.0
11–20	29	12.7
21–30	31	13.6
31–40	35	15.4
41–50	30	13.2
51–60	17	7.5
61–70	24	10.5
>70	19	8.3
**Sex**		
Male	116	51.1
Female	111	48.8
M to F ratio	1:0.9	
**Clinical samples**		
Urine	76	33.4
Pus swabs	57	25.1
Blood	53	23.3
Catheter tips	21	9.2
Sputum	15	6.6
Tissue	5	2.2
**Clinical isolates**		
*Klebsiella pneumoniae*	145	63.8
*Escherichia coli*	82	36.1

### 3.2 Bacterial Isolates in Samples

Of the 227 clinical isolates of GNRs, 82 (36%) were *E. coli* and 145 (63.8%) were *K. pneumoniae*. Seventy‐six (33.4%) isolates were found in urine samples, 57 (25.1%) in pus samples, 53 (23.3%) in blood culture, and 21 (9.2%) on endotracheal tube tips. The isolation rates of *K. pneumoniae* were 29.6% for pus samples, 24.1% for urine, and 23.4% for blood. However, among *E. coli* isolates, 41 were found in urine samples (50.0%), 19 in blood samples (23.1%), and 14 in pus samples (17.0%) (Table 3).

**TABLE 3:** Distribution of clinical isolates across various sample types.

Isolates	Urine	Pus	Blood	ETT tips	Sputum	Tissue
*Klebsiella pneumoniae* (145; 63.8%)	35 (24.1%)	43 (29.6%)	34 (23.4%)	13 (8.9%)	15 (10.3%)	5 (3.4%)
*Escherichia coli* (82; 36.1%)	41 (50.0%)	14 (17.0%)	19 (23.1%)	8 (9.7%)	0 (0%)	0 (0%)
Total	76 (33.4%)	57 (25.1%)	53 (23.3%)	21 (9.2%)	15 (6.6%)	5 (2.2%)

Abbreviation: ETT tips, endotracheal tube tips.

### 3.3 Antibiogram of *Escherichia coli* Clinical Isolates

Of the 227 samples of *E. coli* analyzed in this study, 82 (36.1%) were subjected to MIC testing against different AWaRe classes of antibiotics. Fifty‐eight (70.7%) of the 82 samples produced ESBL. This study found that resistance was prevalent in all isolates to various types of antibiotics, including penicillins (specifically ampicillin), beta‐lactam inhibitors (co‐amoxiclav and piperacillin/tazobactam), and third–fourth‐generation cephalosporins (ceftriaxone, ceftazidime, and cefepime). In addition, over 60% of the GNRs were resistant to quinolones, fluoroquinolones, and tobramycin. The study further found that 46% of the isolates were resistant to fosfomycin and 29% to nitrofurantoin. Furthermore, AMR against amikacin was found in 17.2% of the isolates. However, carbapenems and polymyxin B were the most effective pharmaceuticals for treating these isolates.

**FIGURE 1:**
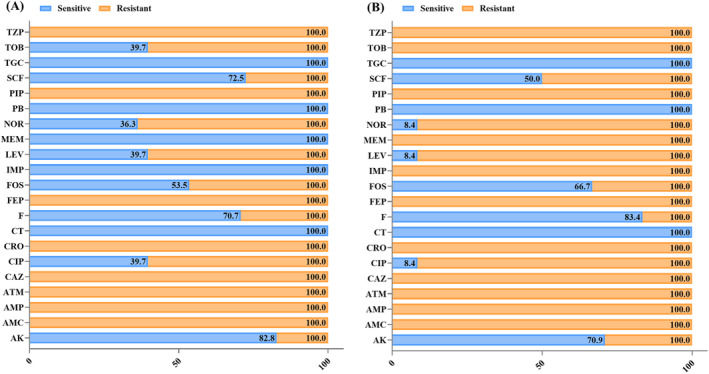
Antibiogram of *Escherichia coli* clinical isolates with color‐coded zones highlighting their antibiotic sensitivity or resistance. (A) The AMR profile of ESBLs; (B) The AMR pattern of carbapenem‐resistant isolates. AK, amikacin; AMC, amoxicillin/clavulanic acid; AMP, ampicillin; ATM, aztreonam; CAZ, ceftazidime; CIP, ciprofloxacin; CRO, ceftriaxone; CT, colistin; F, nitrofurantoin; FEP, cefepime; FOS, fosfomycin; IMP, imipenem; LEV, levofloxacin; MEM, meropenem; NOR, norfloxacin; PB, polymyxin B; PIP, piperacillin; TGC, tigecycline; TOB, tobramycin; TZP, piperacillin/tazobactam.

**FIGURE 2:**
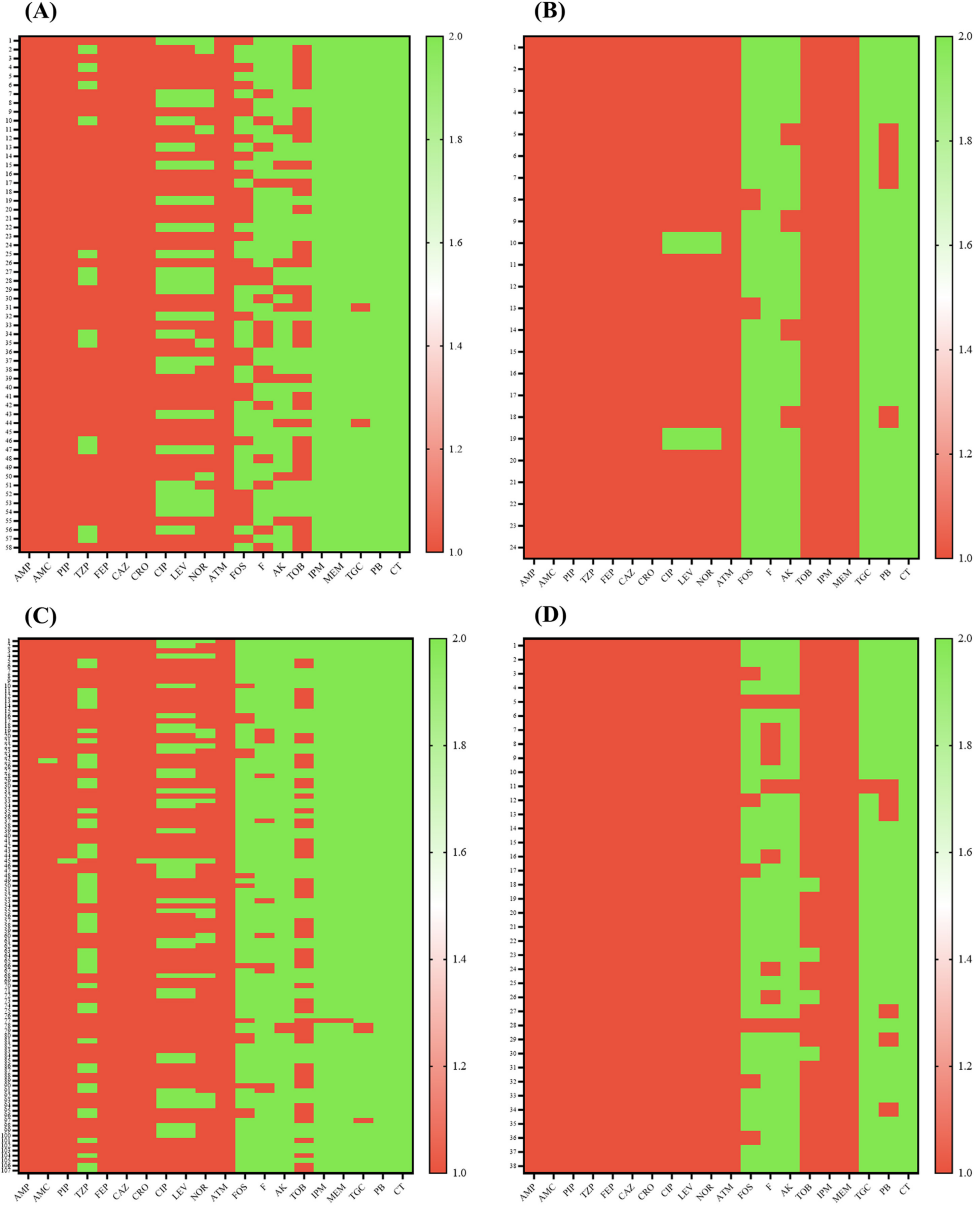
Heatmap presents a comparative analysis of antibiotic sensitivity patterns in ESBL‐producing and CRE *E. coli* and *K. pneumoniae*, using red to show resistance (“1”) and green for sensitivity (“2”). (A) The resistance profile of ESBL‐producing *E. coli*, with a notable red hue, indicates widespread resistance; (B) The resistance profile of CRE *E. coli*, where the red expanse similarly denotes a comprehensive resistance to the antibiotics assessed; (C) ESBL‐producing *K. pneumoniae* exhibits a more heterogeneous pattern, reflected by an interspersion of red and green, with the latter suggesting retained sensitivity to certain antibiotics; (D) The resistance landscape for CRE *K. pneumoniae* illustrates the dominant resistance. AK, amikacin; AMC, amoxicillin/clavulanic acid; AMP, ampicillin; ATM, aztreonam; CAZ, ceftazidime; CIP, ciprofloxacin; CRO, ceftriaxone; CT, colistin; F, nitrofurantoin; FEP, cefepime; FOS, fosfomycin; IMP, imipenem; LEV, levofloxacin; MEM, meropenem; NOR, norfloxacin; PB, polymyxin B; PIP, piperacillin; TGC, tigecycline; TOB, tobramycin; TZP, piperacillin/tazobactam.

Carbapenem resistance was found in 24 of the 82 (29.2%) isolates tested. Ampicillin, cephalosporins, carbapenems, and beta‐lactam inhibitors, such as co‐amoxiclav acid and piperacillin/tazobactam, were completely ineffective against the pathogens tested in this study. Over 90% of the CRE population showed resistance to quinolones and fluoroquinolones. The frequency of amikacin resistance was 29% and that of fosfomycin resistance was 33%. According to the results of this study (Figure 1A,B), colistin and polymyxin B are the two most effective antibiotics in this study. The widespread resistance observed in each bacterial isolate of both ESBL‐producing and CRE *E. coli* underscores a comprehensive analysis of antibiotic resistance patterns (Figure 2A,B).

### 3.4 Antibiogram of *Klebsiella pneumoniae*

**FIGURE 3:**
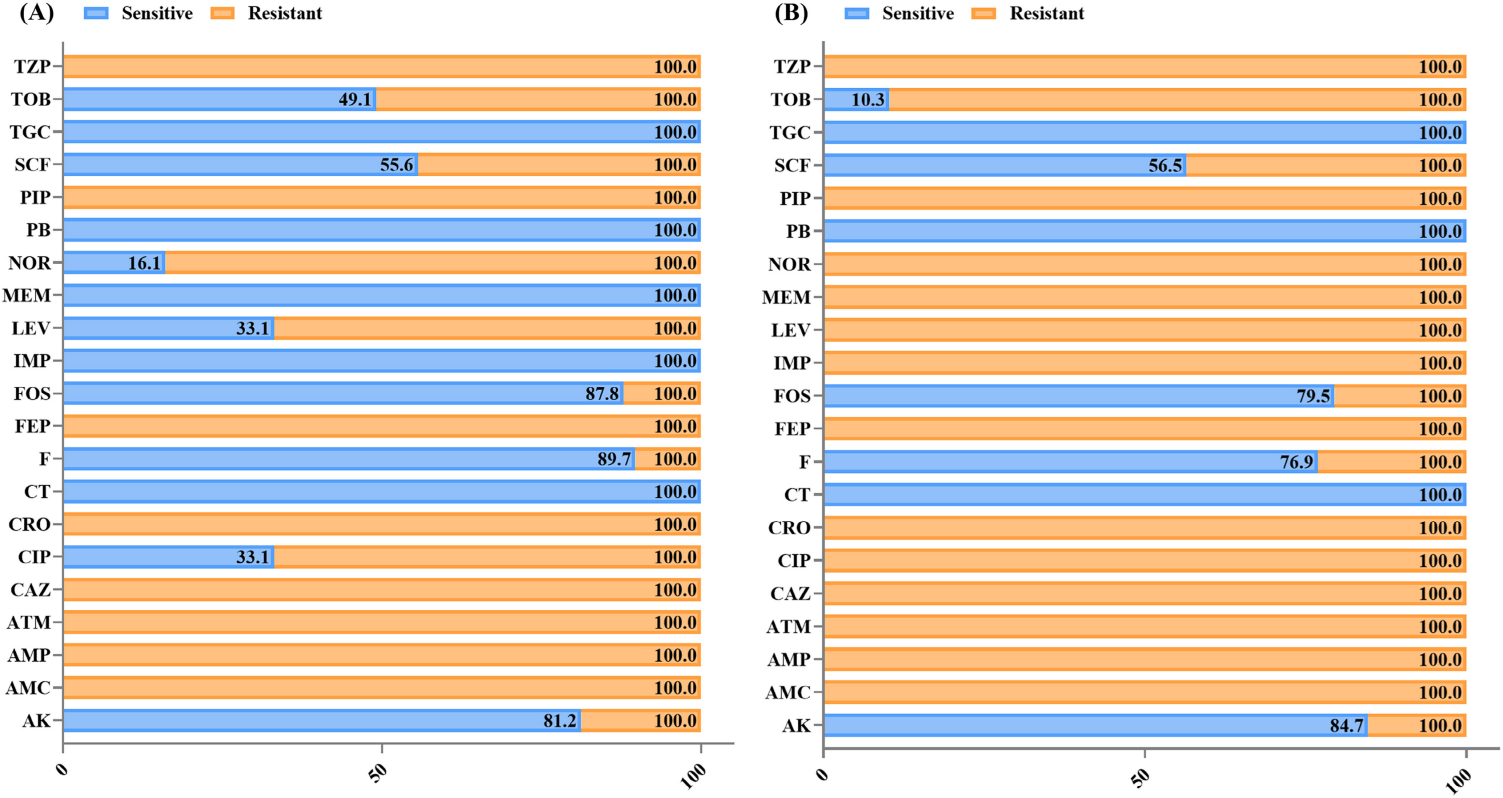
Antibiogram of *Klebsiella pneumoniae* clinical isolates, featuring color‐coded zones to underscore their antibiotic sensitivity or resistance. (A) The AMR characteristics of ESBL producers; (B) the AMR profile of carbapenem‐resistant isolates. AK, amikacin; AMC, amoxicillin/clavulanic acid; AMP, ampicillin; ATM, aztreonam; CAZ, ceftazidime; CIP, ciprofloxacin; CRO, ceftriaxone; CT, colistin; F, nitrofurantoin; FEP, cefepime; FOS, fosfomycin; IMP, imipenem; LEV, levofloxacin; MEM, meropenem; NOR, norfloxacin; PB, polymyxin B; PIP, piperacillin; TGC, tigecycline; TOB, tobramycin; TZP, piperacillin/tazobactam.

Of the 227 clinical isolates of GNRs, 145 (63.8%) were identified as *K. pneumoniae*. The MIC was determined for various medically important antibiotics. Of the 145 (63.8%) isolates of *K. pneumoniae*, 106 (73.1%) were identified as ESBL‐producing. The beta‐lactam antibiotics and beta‐lactam inhibitors were all ineffective against the isolates, with the exception of carbapenem. The AMR against tobramycin resistance was found in 50.9%, whereas ciprofloxacin and levofloxacin resistance was found in 66.9% of the isolates. In addition, 12.2% bacterial resistance to fosfomycin, while 18.8% presented resistance to amikacin. Carbapenems and colistin were effective against the vast majority of the isolates. Thirty‐eight (26.2%) of the isolates were resistant to carbapenem antibiotics, specifically imipenem and meropenem. The *K. pneumoniae* isolates exhibited AMR to a broad range of antibiotics, including beta‐lactams, beta‐lactam inhibitors, and quinolones; 23.1% of the isolates were resistant to nitrofurantoin, whereas 20.5% were resistant to fosfomycin. All of the isolates exhibited sensitivity to colistin and tigecycline (Figure 3A,B). The contrasting resistance patterns observed in each bacterial isolate of both ESBL‐producing and CRE *K. pneumoniae* highlight significant antibiotic resistance, with differences in sensitivity across strains (Figure 2C,D).

### 3.5 Molecular Detection of Resistance Genes in *Escherichia coli*

The PCR technique was employed for identifying CR and ESBL‐producing *E. coli* using targeted primers. The screening process involved the detection of the three most commonly observed ESBL genes, *bla*_CTX‐M_, *bla*_TEM_, and *bla*_SHV_. Among the 82 out of 227 (36.1%) MDR *E. coli* isolates, 58 (70.3%) exhibited a phenotype positive for ESBL. Within this subset, 12 (20.6%) isolates tested positive for the *bla*_CTX‐M_ gene, 11 (18.9%) for the *bla*_TEM_ gene, and 3 (5.1%) for the *bla*_SHV_ gene. Moreover, the *bla*_CTX‐M_ and *bla*_TEM_ genes were found to coexist in nine (15.5%) *E. coli* isolates, whereas three (5.1%) isolates harbored both *bla*_CTX‐M_ and *bla*_SHV_ genes. In addition, five (8.6%) isolates were identified to possess *bla*_CTX‐M_, *bla*_TEM_, and *bla*_SHV_ genes simultaneously. Out of the 82 *E. coli* isolates, 24 (29.2%) exhibited resistance to carbapenem antibiotics, specifically imipenem and meropenem. Eight (33.3%) *E. coli* possessed the *bla*_NDM_ gene, whereas two (8.3%) isolates were found to harbor both *bla*_VIM_ and *bla*_IMP_. The *bla*_NDM_ and *bla*_VIM_ genes coexisted in five (20.8%) *E. coli* isolates, whereas the *bla*_NDM_ and *bla*_IMP_ genes coexisted in three isolates (12.5%) (Table 4).

**TABLE 4:** Profiles of ESBL and CR genes in *Escherichia coli* isolates with antibiotic resistance patterns.

No. of isolates	Ceftriaxone and ceftazidime μg/mL	ESBL gene	No. of isolates	Imipenem and meropenem μg/mL	CR genes
12	≥64	*bla*_CTX‐M_	8	≥16	*bla*_NDM_
11	≥64	*bla*_TEM_	5	≥16	*bla*_NDM_, *bla*_VIM_
3	≥64	*bla*_SHV_	3	≥16	*bla*_NDM_, *bla*_IMP_
9	≥64	*bla*_CTX‐M_, *bla*_TEM_	2	≥16	*bla*_VIM_
3	≥64	*bla*_CTX‐M_, *bla*_SHV_	2	≥16	*bla*_IMP_
3	≥64	*bla*_TEM_, *bla*_SHV_	—	—	—
1	≥64	*bla*_TEM_, *bla*_SHV_	—	—	—
5	≥64	*bla*_CTX‐M_, *bla*_TEM_, *bla*_SHV_	—	—	—

### 3.6 Molecular Detection of Resistance Genes in *Klebsiella pneumoniae*

Of 227 *K. pneumoniae*, 145 (63.8%) were tested positive for ESBL production. Of these ESBL‐positive isolates, 107 (73.7%) exhibited phenotypic characteristics indicating ESBL production. Further examination showed that among these 107 bacteria, 46 (52.3%) carried the *bla*_CTX‐M_ gene, 27 (18.6%) carried the *bla*_TEM_ gene, and 10.3% carried the *bla*_SHV_ gene. Moreover, *bla*_CTX‐M_ and *bla*_TEM_ coexisted in 12 (11.2%) isolates, whereas *bla*_CTX‐M_ and *bla*_SHV_ coexisted in 8 (7.4%) isolates. Thirty‐eight (26.2%) of the 145 *K. pneumoniae* isolates were CR. Twenty‐six (68.4%) of these CR isolates were found to harbor the *bla*_NDM_ gene. The *bla*_NDM_ and *bla*_VIM_ genes coexisted in 13 (34.2%) isolates, whereas *bla*_NDM_ and *bla*_OXA‐48_ genes co‐occurred in 2 (5.2%) isolates (Table 5).

**TABLE 5:** Antibiotic resistance in *Klebsiella pneumoniae* isolates with ESBL and CR genes.

No. of isolates	Ceftriaxone and ceftazidime μg/mL	ESBL gene	No. of isolates	Imipenem and meropenem μg/mL	CR genes
56	≥64	*bla*_CTX‐M_	26	≥16	*bla*_NDM_
27	≥64	*bla*_TEM_	1	≥16	*bla*_VIM_
15	≥64	*bla*_SHV_	1	≥16	*bla*_IMP_
12	≥64	*bla*_CTX‐M_, *bla*_TEM_	13	≥16	*bla*_NDM_, *bla*_VIM_
8	≥64	*bla*_CTX‐M_, *bla*_SHV_	7	≥16	*bla*_NDM_, *bla*_IMP_
4	≥64	*bla*_CTX‐M_, *bla*_TEM_, *bla*_SHV_	2	≥16	*bla*_NDM_, *bla*_OXA‐48_
			1	≥16	*bla*_NDM_, *bla*_VIM_, *bla*_IMP_

## 4 Discussion

The escalating problem of AMR poses a significant worldwide challenge, impacting human health. The widespread and inappropriate application of antibiotics within healthcare facilities, veterinary practices, and the agricultural sector contributes significantly to the worldwide proliferation of AMR. In addition, inadequate sanitation, currency notes, and disposal systems in densely populated areas pose a significant risk of transmission of AMR throughout the community [22, 23]. MDR infections are a major contributor to rising healthcare expenditures, patient death, and length of stay in hospital. It is recommended that antibiotic susceptibility testing of microorganisms, conducted through the use of antibiograms, is performed annually as a minimum requirement. Regular revision of hospital empirical antibiotic policies should be based on this practice [11].

*Escherichia coli* and *K. pneumoniae* are notably prevalent in healthcare environments and frequently identified in various patient‐derived samples. Our findings indicate a higher presence of these pathogens in pediatric patients below the age of 10 years, suggesting possible nosocomial infections linked to their developing immune system. In contrast, patients over 60 predominantly showed ESBL‐producing *E. coli* infections of the urinary tract, similar to the findings of a study from Saudi Arabia on the same subject [24]. Notably, in our study, the primary sources of the samples were urine, pus, and blood. In comparison, a Nepalese study highlighted sputum as the predominant source of *K. pneumoniae*, which were also found in urine and pus [25]. Consistent with earlier studies, a significant association exists between *K. pneumoniae* and ventilator‐associated pneumonia [26]. A study from Faisalabad, Pakistan, confirmed the incidence of *E. coli* in pus and urine specimens [27].

These pathogens have shown increasing antibiotic resistance in the WHO AWaRe classes over the past two decades, leaving clinicians with few options for treatment. The growing number of ESBL and CRE pathogens is a major problem on a global scale [28]. The severity of ESBL‐producing Enterobacterales epidemics in hospitals and communities varies widely. In this study, 77% of the *E. coli* were ESBL producers, whereas 29.2% displayed AMR to carbapenems. In the *K. pneumoniae* isolates, 73.1% produced ESBLs, and 26.2% were CR. The CRE bacterial isolates exhibited resistance to beta‐lactams, with over 90% resistant to fluoroquinolones, whereas colistin demonstrated a high effectiveness. Both ESBL‐producing *E. coli* and *K. pneumoniae* showed resistance to beta‐lactam drugs, excluding carbapenem, and presented moderate resistance (17–46%) to amikacin, nitrofurantoin, and fosfomycin. A meta‐analysis focusing on disseminating ESBL producers in Pakistan found that ESBL producers constituted 40% of the isolates [29]. More recently, a study on AMR in Pakistan revealed that 31% of isolates were ESBL producers [6]. There has been a significant increase in ESBL‐producing *E. coli* in Pakistan, rising from 33.7% in 2005 to 60.0% in 2009–2010. This increase was observed in urine samples, with a prevalence of 31.8–62.9%, and in pus samples, with a prevalence of 55.5% [30]. Several studies on ESBL‐producing Enterobacterales from various cities in Pakistan have reported findings similar to this study [31, 32, 33].

An earlier study in Pakistan reported a 9.7% incidence of CRE in various clinical specimens [6]. In addition, a notable 30% incidence of CRE has been found in clinical samples from Karachi [34]. Our findings are similar and show the prominent presence of ESBL genes, specifically *bla*_CTX‐M_, and *bla*_TEM_, along with carbapenem‐resistant genes like *bla*_NDM_ and *bla*_TEM_. Similarly, a study from Lahore found *bla*_CTX‐M_ in 72% of *E. coli* and 82% of *K. pneumoniae* clinical isolates [35]. The *bla*_NDM_ and *bla*_VIM_ genes have disseminated widely in CRE, starting in Lahore [36], Islamabad [37], and Karachi [38].

The reduced sensitivity of bacteria to antibiotics, which were previously highly effective, can be attributed to several factors [39], including overprescription, inadequate sensitivity testing, and excessive dosing. The issue of MDR has become a significant challenge in disease management [40]. It is crucial to maintain precise data on AMR to maintain the effectiveness of empirical therapy in the evolving patterns of AMR and the emergence of MDR organisms [41]. Our study on *E. coli* and *K. pneumoniae* encounters limitations because of the narrow selection of genera and the exclusive focus on ESBL and CRE‐mediated resistance. Moreover, the lack of extensive genomic sequencing limits our insight into the intricate mechanisms of resistance and the genetic framework surrounding the resistance genes.

## 5 Conclusion

This study provides important insights into the widespread occurrence of ESBL‐producing and CRE in Pakistan and highlights their various resistance strategies. The findings reveal the significant presence of ESBL‐producing and CRE in healthcare environments. The clinical isolates of *E. coli* and *K. pneumoniae* mainly contained the *bla*_CTX‐M_ and *bla*_TEM_ ESBL genes as well as the *bla*_NDM_ and *bla*_VIM_ CR genes. MDR *E. coli* and *K. pneumoniae* demonstrated greater sensitivity to colistin, tigecycline, and polymyxin B. Many of these microorganisms have developed resistance to antibiotics. Performance of culture sensitivity assessments is vital for the judicious and knowledgeable use of antibiotics in healthcare settings. This is especially important in light of the growing concerns over AMR. By leveraging up‐to‐date antibiograms to evaluate susceptibility trends, healthcare professionals can make better‐informed choices regarding empirical antibiotic treatments, aiding in the battle against antibiotic resistance.

## Conflicts of Interest

The authors declare no conflicts of interest.

## Data Availability

Data are available on request from the authors.
